# Influence of patient and hospital characteristics on inpatient satisfaction in China's tertiary hospitals: A cross‐sectional study

**DOI:** 10.1111/hex.12974

**Published:** 2019-10-22

**Authors:** LinLin Hu, Hui Ding, Shiyang Liu, Zijuan Wang, Guangyu Hu, Yuanli Liu

**Affiliations:** ^1^ School of Public Health Chinese Academy of Medical Sciences & Peking Union Medical College Beijing China; ^2^ Department of Economics Stanford University Stanford California; ^3^ Institute for Medical Information Chinese Academy of Medical Science Beijing China

**Keywords:** Chinese health care, demographic characteristics, hospital performance, hospital structure, patient satisfaction

## Abstract

**Background:**

Patient satisfaction has been seen as a key criterion when evaluating hospitals and is one of the main focuses of the current health‐care reform in China. This paper aimed to explore patient‐ and hospital‐level factors associated with inpatient satisfaction, which can provide policy implications for the evaluation and development of a patient‐oriented health‐care system.

**Methods:**

The paper analyses data from the 2017 China National Patient Survey which includes 20 300 inpatients from 131 tertiary hospitals across 31 provinces. Descriptive analysis and multivariable logistic regressions are conducted to identify key factors related to satisfaction.

**Results:**

Patient sociodemographic characteristics, including gender, age, income and insurance type, are found to be strongly associated with their satisfaction of inpatient experience. In terms of institutional characteristics, hospital type, size, staffing and financial performance are also significantly correlated with inpatient satisfaction. Patients are more satisfied with specialist hospitals and large hospitals measured by the number of beds and surgeries. Hospitals with higher nurse‐to‐bed ratio also receive more satisfaction. The financial performance of hospitals, however, is negatively associated with satisfaction.

**Conclusion:**

Patient satisfaction contains unique information on service quality and thus should be incorporated into the matrix of hospital evaluation. Meanwhile, differences in patient composition must be adjusted to make fair comparisons across hospitals. Moreover, future reform needs to put greater efforts in the design of comprehensive public insurance scheme, efficient hospital structure and an overall well‐functioning health‐care delivery system in order to better serve patients in China.

## 1 INTRODUCTION

Patient satisfaction has long been recognized as a key measurement of hospital quality. Apart from observable health outcomes, patients' assessments of their experience with health‐care providers can capture unique information on the provision of care, for example, complications that are hard to quantify, patient involvement in treatment decisions and physicians' explanation of procedures. Over the past 20 years, many developed countries have been trying to include patient satisfaction surveys as a quality improvement tool in health‐care markets.^1^, ^2^, ^3^, ^4^ China is also putting a great emphasis on improving the quality of care and patient satisfaction in her current health care system reform.^5^, ^6^ The ‘Healthy China 2030’ initiative raised a set of requirements, which include promoting service appointment systems, optimizing ward structure and enhancing health information system to improve patients' hospital experience.^7^

In order to evaluate the improvement of health‐care quality, Peking Union Medical College School of Public Health was commissioned by the Chinese government as a third‐party academic institution to perform annual surveys of patients, known as the China National Patient Survey.^7^ This survey collects data from 136 tertiary hospitals, which are defined as institutions with more than 500 beds in China. Tertiary hospitals are a major provider of inpatient service in Chinese health‐care system. In 2017, there were 2340 tertiary hospitals nationwide, which accounted for 7.5% of hospitals and served 44.4% of inpatient visits.^8^ Among these tertiary institutions, over 90% are public hospitals which are self‐financing entities responsible for their own balance sheets while receiving some government funding.^8^, ^9^, ^10^ In this paper, we use data collected in 2017 to explore key factors related to inpatient satisfaction, which will help to have a better understanding of how to incorporate patient satisfaction into hospital evaluation and guide future reforms in improving hospital service quality.

There has been a large strand of literature trying to identify the relationship between hospital structure and patient satisfaction in developed countries. Most of these studies have shown that higher staff‐to‐patient ratio and better hospital environment were associated with higher patient satisfaction rates.^11^, ^12^ Large hospitals and teaching hospitals, however, received lower scores.^12^, ^13^, ^14^, ^15^, ^16^, ^17^, ^18^, ^19^

In China, although there has been increasing attention on health‐care quality from both the government and the public, there is a lack of rigorous studies examining how hospital characteristics correlate with inpatient satisfaction. Most of the studies were restricted by the scope of their surveys which only involved a small number of hospitals in a single geographic region.^20^, ^21^, ^22^, ^23^, ^24^, ^25^, ^26^, ^27^, ^28^, ^29^ The focuses of these studies are patient demographic characteristics,^20^, ^21^ insurance type,^22^ expenditure and payment method^20^, ^21^; hospital type,^23^ staffing,^28^, ^29^ number of surgeries^26^; doctors' and nurses' interpersonal interactions,^22^, ^24^ service attitude^20^, ^27^; and the correlation between different dimensions of satisfaction.^27^ Among the few studies that used data from nationwide surveys, it has been shown that higher‐level hospitals, lower competition in providers' market and higher market share of private hospitals were negatively correlated with inpatient satisfaction.^30^ Higher patient‐to‐nurse ratio, however, had ambiguous relationship with patient rating.^31^, ^32^ There are also studies looking at how patient–doctor relationship correlates with patient satisfaction.^7^, ^33^ However, such measurements are more of reflections than the causes of patient satisfaction. For example, patients tend to have more trust towards their doctors when they feel satisfied with their treatment experience. Therefore, enhancing patient–doctor relationship is more like setting a goal instead of providing practical suggestions for hospitals to improve service quality.

It is also a concern that most of papers discussing relationship between hospital structure and patient satisfaction used correlation analysis without controlling for patient characteristics. Since it has been shown in both foreign and Chinese settings that patients' age, insurance type and health status have strong relations with their evaluation,^14^, ^16^, ^20^, ^21^, ^22^, ^30^, ^34^ such correlation studies might be biased by omitted patient‐level factors. For example, if old patients are more likely to visit large hospitals and also tend to rate high in satisfaction surveys, there would be a positive correlation between large hospital and satisfaction score, while we can hardly conclude from it that larger hospitals have better services.

By including both patient‐ and hospital‐level variables into regression analysis, we are able to explore key factors associated with satisfaction holding other confounding factors from the other side constant. The results of both sides can also have policy implications of two directions. On the one hand, our results on the relationship between patient characteristics and satisfaction can help to construct future hospital evaluation matrix which adjust for differences in patient composition across hospitals. Major institutional factors identified in this study, on the other hand, will provide guidance for the improvement of hospital quality and promote the development of a patient‐oriented health‐care system.

## 2 METHODS

### 2.1 Data

This study uses data from the 2017 China National Patient Survey which was collected from 136 tertiary hospitals across 31 provinces during December 2017–January 2018. In order to take into account different types of hospitals, one provincial general hospital, one provincial traditional Chinese medicine (TCM) hospital, and one maternal and child health hospital were selected from each province. In addition, 43 hospitals affiliated with National Health and Family Planning Commission, including 28 general hospitals and 15 specialist hospitals, were also included. All hospitals in the sample are public hospitals.

Both inpatients and outpatients were interviewed in the survey. However, in this paper, we only focus on inpatient satisfaction and factors related to it. At least 150 inpatient respondents who were to be discharged on the survey days were selected from each hospital (for hospitals with not sufficient discharges, discharged patients were all selected for the survey; for hospitals with sufficient discharges, patients were stratified by specialties/wards), generating a total of 21 125 respondents among which 21 092 were effective respondents. The interviews were conducted on‐site in the wards by a group of pre‐trained medical students. The inpatient questionnaire has five domains, including Process management, Hospital environment, Nursing care, Physician care and Overall satisfaction (detailed contents are listed in Table 1). A total of 20 questions were asked using a Likert scale from 1 through 5, corresponding to ‘very unsatisfied’, ‘unsatisfied’, ‘neutral’, ‘satisfied’ and ‘very satisfied’, respectively. The questionnaire was validated by small‐scale multidisciplinary expert consultations, patients' cognitive interviews and pilot field tests. Information on patients' characteristics, such as age, gender, education, income, insurance type and length of stay, was also collected.

**Table 1:** Content of the five domains, internal consistency and average domain score

Domain	Items	Content	Cronbach's *α*	Average score
Process management	3	‐ Waiting time for admission ‐ Check‐in procedure ‐ Channel available to complement or complain	.753	4.68
Hospital environment	5	‐ Quietness of the ward ‐ Quality of meals ‐ Accompany of disabled patients by hospital personnel to get tests ‐ Facilities to prevent falling ‐ Convenience to use the elevators	.817	4.56
Nursing care	5	‐ Attitude of nurses ‐ Skills of nurses ‐ Timely help provided by nurses ‐ Nurse in charge of my bed is responsible and careful ‐ Care by nurse aid	.904	4.77
Physician care	5	‐ Inquiry of symptoms with patience ‐ Explanation of treatment with patience ‐ Engagement of patient in decision making ‐ The physician in charge of my bed is responsible and careful ‐ Physician's medical skills	.913	4.77
Overall satisfaction	2	‐ Overall I am satisfied with this stay ‐ I would recommend this hospital to my family and friends	.812	4.70

Hospital‐level information includes number of doctors, nurses, beds, total cost and revenue in 2017. These data were collected from the statistical department of the hospital.

### 2.2 Analysis

Due to missing data for cost/revenue, five hospitals are excluded from the sample, together with 792 patients from these hospitals, leaving a sample of 131 hospitals and 20 300 patients. For each of the five satisfaction domains, we calculate the average score of the included items as domain‐level satisfaction score and test for internal consistency using Cronbach's *α* coefficient. As shown in Table 1, the average satisfaction scores range from 4.56 to 4.77, with Cronbach's *α* all at acceptable level.

In order to show the relationship between patient characteristics, hospital characteristics and the satisfaction score in each domain, we first present descriptive evidence using Kruskal‐Wallis and Mann‐Whitney tests for difference in satisfaction scores between different sociodemographic groups and hospital categories. In order to focus more on the factors associated with patients being satisfied, we then proceed to multivariable logistic regression where we translate satisfaction scores for each domain into binary variables which equal to 1 if the score is higher than 4 (indicating ‘satisfied’ or ‘very satisfied’ in the original questionnaire) and 0 otherwise. Independent variables of interest including age, gender, education, income category, insurance type, length of stay of each inpatient, and specialty type, number of beds, number of surgeries, doctor‐to‐nurse ratio, nurse‐to‐bed ratio, revenue‐to‐cost ratio of each hospital were all put in the regressions using enter method. All data analysis was performed using SPSS version 22.0.

## 3 RESULTS

### 3.1 Descriptive statistics

Summary statistics for patient characteristics are presented in Table 2. Among 20 300 respondents, 40.62% were male (48.94% if exclude patients in maternal and child health hospital) with an average age of 47.7. The household income is grouped into below and above 60 000 RMB, each accounting for 35.6% and 64.6% of the sample. 36.1% held an education level of middle school or below, and 63.9% were high school or above. Insurance type includes three major public insurance: Urban Employee Basic Medical Insurance (UEBMI) for individuals employed in the formal sectors in cities, New Rural Cooperative Medical Insurance (NRCMI) for rural residents (defined by household registration status) and Urban and Rural Resident Basic Medical Insurance (RBMI) which covers urban residents who are unemployed or in informal sectors, and rural residents in regions that have integrated NRCMI with the previous Urban Resident Basic Medical Insurance. Among the survey sample, 31.21% of the inpatients hold UEBMI, while 22.71% and 29.78% are covered by RBMI and NRCMI, respectively. Beyond that, there are 12.37% of the patients under Government Insurance Scheme (GIS, eligible for government officials and employees), leaving the rest 3.93% patients uninsured. In terms of the length of stay, around half of the sampled patients stayed in hospital for 7 days or less.

**Table 2:** Patient characteristics and mean domain scores for each group

	Number of Patients (%)	Process management	Hospital environment	Nursing care	Physician care	Overall satisfaction
Gender						
Male	8246 (40.62%)	4.68	4.56	4.77	4.77	4.69
Female	12 054 (59.38%)	4.68	4.55	4.77	4.77	4.70
*P*		.985	.468	.408	.300	.022
Age						
18‐35	6844 (33.71%)	4.66	4.53	4.76	4.75	4.67
35‐65	9580 (47.19%)	4.68	4.57	4.78	4.78	4.70
>35	3876 (19.09%)	4.70	4.59	4.78	4.79	4.72
*P*		.001	<.001	.001	<.001	<.001
Income						
0‐60 000 RMB	7227 (35.60%)	4.63	4.51	4.73	4.73	4.65
>60 000 RMB	13 073 (64.40%)	4.71	4.58	4.79	4.79	4.72
*P*		<.001	<.001	<.001	<.001	<.001
Education						
Middle school or below	7328 (36.10%)	4.66	4.55	4.76	4.76	4.68
High school or above	12 972 (63.90%)	4.69	4.56	4.78	4.78	4.70
*P*		.009	.152	.017	.003	.004
Insurance						
GIS	2424 (12.37%)	4.76	4.65	4.83	4.84	4.77
UEBMI	6115 (31.21%)	4.70	4.57	4.79	4.79	4.72
RBMI	4450 (22.71%)	4.66	4.54	4.75	4.75	4.67
NRCMI	5835 (29.78%)	4.64	4.52	4.75	4.75	4.66
No insurance	770 (3.93%)	4.69	4.56	4.79	4.77	4.71
*P*		<.001	<.001	<.001	<.001	<.001
Length of stay						
≤7	10 335 (50.91%)	4.68	4.57	4.78	4.77	4.70
>7	9965 (49.09%)	4.67	4.55	4.76	4.77	4.69
*P*		.039	.002	.001	.193	.022

Abbreviations: GIS, Government Insurance Scheme; NRCMI, New Rural Cooperative Medical Insurance; RBMI, Urban and Rural Residents Basic Medical Insurance; UEBMI, Urban Employees Basic Medical Insurance.

Mann‐Whitney tests for satisfaction score between different patient groups show that elder patients are more satisfied in all five dimensions. Higher income and education level are also correlated with higher satisfaction score in most of the domains. Patients under different insurance types are also proved to be significantly different in average satisfaction scores under Kruskal‐Wallis test, with the uninsured having lowest satisfaction and the government‐insured highest. Shorter inpatient stay, indicating better health or less complicated conditions, is associated with better satisfaction rating.

Similar as patient characteristics, Table 3 summarizes hospital characteristics and the differences in satisfaction scores between hospital groups. Hospital types are categorized as general hospitals (41.22%), traditional Chinese medicine hospital (TCM, 22.9%), maternal and child health hospital (22.14%) and specialist hospitals (13.74%). Kruskal‐Wallis test shows significant differences in all five satisfaction scores, with general and specialist hospitals receiving higher patient satisfaction compared to the other two groups. Hospital size is measured by the number of beds and number of surgeries performed in 2017. Direct comparisons between large and small hospitals are made by dividing the sample by the median, that is 1400 beds and 22 154 surgeries per year. Both of the tests show that larger hospitals have higher score in each of the satisfaction domains. Doctor‐to‐nurse ratio and nurse‐to‐bed ratio are also grouped as above or below the median. However, while hospitals with lower doctor‐to‐nurse ratio only have slightly higher overall score and satisfaction towards process management, higher nurse‐to‐bed ratio is strongly associated with higher satisfaction by all five measurements. Finally, in terms of hospital financial performance, we compare hospitals with revenue‐to‐cost ratio above and below 1, or in other words, hospitals with financial gain or loss. Mann‐Whitney tests indicate that profitable hospitals receive lower score in either satisfaction domain.

**Table 3:** Difference in mean domain scores according to hospital characteristics

	Number of hospitals (%)	Process management	Hospital environment	Nursing care	Physician care	Overall satisfaction
Hospital type						
General	54 (41.22%)	4.70	4.60	4.79	4.79	4.72
TCM	30 (22.90%)	4.66	4.50	4.74	4.75	4.65
Maternal	29 (22.14%)	4.67	4.53	4.75	4.74	4.67
Specialist	18 (13.74%)	4.64	4.58	4.80	4.81	4.73
*P*		<.001	<.001	<.001	<.001	<.001
# of beds						
≤1400	66 (50.38%)	4.63	4.51	4.73	4.73	4.65
>1400	65 (49.62%)	4.73	4.61	4.80	4.81	4.74
*P*		<.001	<.001	<.001	<.001	<.001
# of surgeries per year						
≤22 154	66 (50.38%)	4.62	4.48	4.71	4.71	4.62
>22 154	65 (49.62%)	4.73	4.63	4.83	4.82	4.77
*P*		<.001	<.001	<.001	<.001	<.001
Doctor‐to‐nurse ratio						
≤0.63	63 (48.09%)	4.68	4.56	4.77	4.77	4.70
>0.63	68 (51.91%)	4.67	4.55	4.77	4.77	4.69
*P*		.022	.306	.703	.730	.040
Nurse‐to‐ bed ratio						
≤0.72	66 (50.38%)	4.67	4.53	4.76	4.76	4.68
>0.72	65 (49.62%)	4.68	4.58	4.78	4.78	4.71
*P*		.007	<.001	<.001	<.001	<.001
Revenue‐to‐cost ratio						
<1	26 (19.85%)	4.71	4.60	4.78	4.79	4.72
≥1	105 (80.15%)	4.67	4.55	4.77	4.77	4.69
*P*		<.001	<.001	.002	.001	<.001

### 3.2 Patient characteristics

Now we turn to multivariable logistic regression to explore key characteristics related to patient satisfaction. From the patient side, as shown in Table 4, gender, age, income level and insurance type all show strong relationship with various of satisfaction scores, while education level and length of stay are not significantly correlated after controlling other patient and hospital characteristics.

**Table 4:** Multivariable logistic regression of patient satisfaction on patient and hospital characteristics (OR with 95% confidence interval)

	Specification	Process management	Hospital environment	Nursing care	Physician care	Overall satisfaction
Gender	Male (reference)					
	Female	1.039 (0.914,1.182)	1 (0.913,1.095)	1.05 (0.891,1.236)	1.178 (0.989,1.403)	1.164* (1.007,1.346)
Age	18‐35 (reference)					
	35‐65	1.04 (0.896,1.208)	1.098 (0.987,1.221)	1.23* (1.013,1.494)	1.398* (1.144,1.707)	1.151 (0.972,1.363)
	>65	1.265* (1.028,1.556)	1.207* (1.045,1.393)	1.232 (0.954,1.59)	1.755** (1.323,2.327)	1.476* (1.166,1.869)
Income	0‐60 000 RMB (reference)					
	>60 000 RMB	1.342** (1.177,1.53)	1.26** (1.149,1.383)	1.484** (1.257,1.753)	1.318* (1.104,1.573)	1.372** (1.184,1.59)
Education	Middle school and below (reference)					
	High school and up	1.023 (0.887,1.18)	0.976 (0.882,1.081)	0.992 (0.826,1.19)	1.131 (0.932,1.372)	0.876 (0.745,1.032)
Insurance	GIS (reference)					
	UEBMI	0.868 (0.688,1.097)	0.695** (0.593,0.815)	0.783 (0.581,1.055)	0.74* (0.533,1.027)	0.809 (0.625,1.048)
	RBMI	0.678** (0.535,0.86)	0.648** (0.549,0.766)	0.654* (0.481,0.888)	0.576* (0.414,0.803)	0.68* (0.521,0.887)
	NRCMI	0.643** (0.506,0.818)	0.662** (0.559,0.785)	0.65* (0.477,0.887)	0.644* (0.459,0.904)	0.649* (0.495,0.85)
	No insurance	0.827 (0.571,1.198)	0.745* (0.574,0.967)	1.149 (0.672,1.965)	0.908 (0.533,1.547)	1.067 (0.676,1.683)
Length of stay	≤7 (reference)					
	>7	0.972 (0.855,1.105)	0.987 (0.901,1.081)	0.923 (0.782,1.089)	0.984 (0.826,1.171)	0.924 (0.798,1.069)
Hospital type	General (reference)					
	TCM	1.67** (1.36,2.05)	1.024 (0.886,1.183)	1.421* (1.11,1.818)	1.938** (1.48,2.537)	1.257* (1.007,1.57)
	Maternal	1.615** (1.298,2.009)	1.25* (1.065,1.466)	1.338* (1.015,1.762)	1.419* (1.072,1.879)	1.233 (0.965,1.575)
	Specialist	1.253 (1,1.57)	1.332* (1.12,1.584)	1.731* (1.245,2.406)	2.435** (1.694,3.501)	1.538* (1.156,2.047)
# of beds	≤1400 (reference)					
	>1400	1.737** (1.468,2.057)	1.381** (1.228,1.552)	1.285* (1.051,1.57)	1.532** (1.227,1.912)	1.284* (1.075,1.533)
# of surgeries per year	≤22 154 (reference)					
	>22 154	1.495** (1.289,1.734)	1.357** (1.22,1.508)	2.14** (1.751,2.616)	2.013** (1.637,2.474)	2.219** (1.857,2.652)
Doctor‐to‐nurse ratio	≤0.63 (reference)					
	>0.63	1.082 (0.949,1.234)	1.124* (1.023,1.234)	0.97 (0.817,1.15)	1.078 (0.902,1.289)	1.017 (0.874,1.184)
Nurse‐to‐bed ratio	≤0.72 (reference)					
	>0.72	1.057 (0.917,1.219)	1.108* (1.001,1.227)	1.28* (1.06,1.545)	1.26* (1.04,1.525)	1.251** (1.058,1.479)
Revenue‐to‐cost ratio	<1 (reference)					
	≥1	0.735** (0.623,0.867)	0.751** (0.67,0.842)	0.775* (0.63,0.953)	0.82 (0.663,1.015)	0.748* (0.622,0.901)
% Correct		94.1	87.1	96.5	96.8	95.4
likelihood ratio (*χ* ^2^)		201.569 (*P* < .001)	225.371 (*P* < .001)	180.784 (*P* < .001)	177.985 (*P* < .001)	237.688 (*P* < .001)

* *P* < .05.

** *P* < .01.

In terms of gender, female patients are more likely to have higher overall satisfaction score (OR = 1.164). However, there is no statistically significant difference in separate satisfaction domains between male and female patients. As for age, compared to younger patients under age 35, elder patients are more satisfied in all dimensions, especially for age group above 65 (OR = 1.476). Higher income also predicts higher satisfaction scores. The odds ratios for patients with household income over 60 000 RMB to be satisfied, compared to lower income patients, exceed 1.25 for all of the five domains.

Another important patient‐level characteristics are the type of insurance they hold. As shown in Table 4, compared to patients under Government Insurance Scheme (GIS), patients with either Resident Basic Medical Insurance (RBMI) or New Rural Cooperative Medical Insurance (NRCMI) are less satisfied in all parts of their inpatient experience (overall satisfaction: OR = 0.68 for RMBI; 0.649 for NRCMI). Urban employees under Urban Employee Basic Medical Insurance (UEBMI) also have lower odds of being satisfied, although the difference is relatively smaller and only statistically significant in satisfaction score towards hospital environment. Surprisingly, uninsured patients show neither consistently nor significantly lower satisfaction rate compared to patients under GIS. However, given the extremely small share of uninsured patients in the sample, it is hard to make any credible inference from this group of correlation.

### 3.3 Hospital characteristics

From the institutional side, factors of interest include hospital type, size, personnel structure and financial performance. When compared to general hospitals, all the other three types of hospitals are more likely to receive higher scores in all five satisfaction measurements. The odds ratio for overall satisfaction is largest for specialist hospitals (OR = 1.538), followed by traditional Chinese medicine (OR = 1.257) and then maternal hospitals (OR = 1.233). This is opposite with the comparison made in Table 3, where there is no control for other hospital‐ and patient‐level characteristics. In terms of specific dimensions, the largest difference occurs in satisfaction towards physician care, suggesting one of the critical improvements that general hospitals should focus on in the future.

Two measurements of hospital size, number of beds and number of surgeries, are both strongly associated with satisfaction scores. Large hospitals, categorized as above the sample medians, have OR ratios of 1.284 and 2.219 in overall satisfaction compared to their smaller counterparts. Based on measurements of personnel structure, hospitals are also divided into two groups in order to have better interpretation of the result. Inpatients tend to prefer higher doctor‐to‐nurse ratio (OR = 1.017 for overall satisfaction) and nurse‐to‐bed ratio (OR = 1.251 for overall satisfaction), although the former is not statistically significant at 95% confidence interval. Besides overall satisfaction, higher nurse‐to‐bed ratio is associated with higher satisfaction with nursing care (OR = 1.280) and physician care (OR = 1.26). Hospital financial performance and patients' satisfaction also show strong correlation. As presented in Table 4, hospitals with financial profits are earning significantly lower satisfaction score across all dimensions (OR = 0.748 for overall satisfaction).

The model performs well in terms of prediction and goodness of fit, with the per cent of correctness higher than 85% and chi‐square *P*‐values smaller than .001 for all dimensions. To check the robustness, we also use average length of stay, number of beds and doctor‐to‐nurse ratio in tertiary hospitals collected from National Health Statistical Yearbook^8^ as cut‐offs, and the regression results remain the same.

## 4 DISCUSSION

Improving patient satisfaction has been emphasized as one of the main objectives in the current reform of China's health‐care system. While devoting great efforts into developing patient‐oriented delivery system, we need to have a better understanding on what is associated with patient satisfaction, how to evaluate and what we can do to improve it. In this study, we use descriptive analysis and multivariable logistic regression to explore factors at both patient level and hospital level that are strongly related to patient satisfaction.

From patient side, we find that gender, age, education, income and insurance type are significantly associated with inpatient satisfaction. Since hospitals have a large spread in their patient composition, the evaluation of patient satisfaction across health‐care organizations must take into account differences in these patient characteristics. For example, the share of elder patients (aged over 65) was 27.71%, 31.62%, 0.99% and 14.63%, respectively for general, TCM, maternal and specialist hospitals, indicating large heterogeneity in patients' age structure. Moreover, hospitals in less developed area may face a larger proportion of patients with lower education and income level. A direct comparison of patient satisfaction score between hospitals facing more elder or poor patients and others is unfair and might impede the further improvement of service quality in such hospitals.

Another factor from the patient side that needs to be emphasized is the insurance type. Our results show that patients under Residents Basic Medical Insurance (RBMI) and New Rural Cooperative Medical Insurance (NRCMI) are less satisfied with their inpatient experience compared to patients under Government Insurance Scheme (GIS), even after controlling for patients' income, education and a set of hospital characteristics. The differences in satisfaction between patients under GIS and those under Urban Employees Basic Medical Insurance (UEBMI) are less significant. One possible explanation is the difference in the benefit design across these insurances. While government officials and urban employees enjoy a comprehensive coverage from GIS and UEBMI, the rest of the population are mostly covered by public insurances that are shallow in both reimbursement rate and service list.^35^, ^36^, ^37^ Although commercial insurances are also available, only a small share of people enrols in these plans due to reasons such as high premium, complex design and misperception.^38^ Besides promoting commercial insurances, it is equally, if not more, important to expand the coverage of public insurances to better alleviate financial burdens of patients during negative health shocks.

From hospital side, we found that hospital type, size, personnel structure and financial performance are all strongly correlated with patient satisfaction. Compared to specialist hospitals, general hospitals require more attention during the movement of improving patient satisfaction. In contrast to findings in developed countries, where larger hospitals tend to receive lower patient satisfaction score,^12^, ^13^, ^14^, ^15^, ^16^, ^17^, ^18^, ^19^ patients in China are more likely to rate higher for their stay in larger institutions. Such relationship can have two underlying mechanisms. On the one hand, larger hospitals tend to have more capable physicians, higher capacity in treating complicated conditions and thus better service quality. On the other hand, hospitals with better capacity can attract more patients therefore grow in size, especially measured by the number of surgeries. When looking at specific dimensions of satisfaction, same as hospital type, the largest difference between hospitals with different sizes is found in satisfaction towards physician and nursing care. Actually quality disparity between hospitals is a big issue in China. While top‐tier hospitals can employ highly educated doctors and offer series of advanced procedures, the competency of most physicians in lower‐level hospitals is a big concern, especially in rural area.^39^ As a result, patients are often more willing to self‐refer to higher‐level providers although these hospitals tend to be overcrowded. Besides our study comparing tertiary hospitals of different sizes, there is also literature looking at different levels of hospitals in China, which found that patients in top‐tier hospitals had more unsatisfying experience.^28^ Therefore, policy implications cannot be simply derived as increasing the scale of hospitals since it will aggravate the concentration of medical resources and limit the equal access to quality care. Doctors' training, local hospital quality improvement and the development of an efficient referral system are all essential parts in future reforms of the health‐care system.

Furthermore, patients also appreciate higher nurse‐to‐bed ratio and doctor‐to‐nurse ratio during their inpatient stay. This is consistent with findings in previous studies on the nursing resources in Chinese hospitals, where heavy workload and widespread job burn‐out among nurses are documented as serious threat to quality of care.^28^, ^29^, ^31^, ^32^ This calls for attention on the design of hospital personnel structure. One of the essential problems to be addressed is how to ensure a strong and healthy nurses workforce during the health‐care reform.

Finally, our model finds that hospital financial performance is negatively associated with patient satisfaction. One explanation is that hospitals with financial profits are prescribing more profitable check‐ups or procedures, leading to higher charges which cause patient dissatisfaction. Such result is consistent with previous literature in Canada, where they use the difference between expected and actual cost per case to measure hospital financial efficiency and found slightly negative correlation between this and service quality.^15^ This suggests that high satisfaction of some hospitals might come at the expense of financial efficiency, while the profit of other hospitals is generated by prescribing excessive procedures or drugs regardless of patients' needs. Considering the fact that health service prices are highly regulated in China, it is important to devote more efforts in designing incentive structure for providers to control cost without compromising quality.

When interpreting our findings, several limitations must be acknowledged. First, although this is the first major national study of inpatient satisfaction in China, the survey only focused on tertiary hospitals, and only three hospitals were sampled from each province. Therefore, the result might not be able to generalize to smaller hospitals and less developed areas that do not have such institutions. Second, even with regression that includes factors of both patient and hospital characteristics, we still cannot fully address selection effect of patient into different hospitals. Omitted variables might be correlated with satisfaction, patient sociodemographic measures and the hospital they choose. For example, although we try to include patients' length of stay as a proxy for the severity of disease, it could not fully reflect the complexity and seriousness of the condition. If patients with severe conditions tend to visit larger hospitals and give lower (higher) ratings, such selection would lead to an under‐ (over‐) estimation of the correlation between hospital size and patient satisfaction. Thus, our results cannot be interpreted as casual effects of these factors, and further studies eliminating such confounding factors are still needed to guide future attempts in improving health‐care quality.

## 5 CONCLUSION

This paper explores patient and hospital factors related to inpatient satisfaction. We found that patient gender, age, income and insurance type are associated with their satisfaction towards inpatient stays. Female and elder patients tend to have higher satisfaction rating, as well as patients with higher income and more comprehensive insurance. Hospital type, size, personnel structure and financial performance also have significant correlation with the ratings of patients. Specialist hospitals, large hospitals and those with higher nurse‐to‐bed ratio receive more satisfaction. The financial performance of hospitals, however, is negatively associated with satisfaction. These results suggest that, on the one hand, patient characteristics must be adjusted when incorporating satisfaction into hospital evaluation. On the other hand, future health‐care reform should focus more on designing better public insurance benefits, efficient hospital structure and a well‐functioning health‐care delivery system.

## CONFLICT OF INTEREST

The authors report no conflict of interest in this work.

## AUTHOR CONTRIBUTIONS

LH and HD designed the research question and structure of the paper together. LH and SL performed data cleaning and statistical analyses. HD was the major contributor in writing the manuscript. YL provided guidance and overall support for the paper. All authors read and approved the final manuscript.

## Data Availability

The data that support the findings presented in this study are available from the Public Health School of Peking Union Medical College with a few restrictions. The data were used under licence for the current study and so are not publicly available. Data are, however, available from the authors upon reasonable request and with permission from the Authority of Public Health School of Peking Union Medical College.
